# Brief report: STING expressed in tumor and non-tumor compartments has distinct roles in regulating anti-tumor immunity

**DOI:** 10.1007/s00262-022-03327-w

**Published:** 2022-11-17

**Authors:** Jennie C. Kim, Xian Liu, Karen Fitzgerald, Jason S. Eng, Jessica Orf, Sarah A. O’Brien, Brian Belmontes, Amy-Jo Casbon, Sergey V. Novitskiy, Kristin V. Tarbell, Jason DeVoss, Jackson G. Egen

**Affiliations:** Department of Oncology, Amgen Discovery Research, Amgen Inc., South San Francisco, CA USA

**Keywords:** STING, Tumor immunity, Type I IFN, Immune checkpoint inhibitor

## Abstract

Type I interferon-mediated activation of immune cells can facilitate the generation of productive tumor antigen-specific T cell responses in solid tumors. The cGAS/STING DNA sensing pathway is a critical upstream mediator of type I interferon production and is an important regulator of anti-tumor immunity. Numerous STING pathway agonists are now being tested in clinical trials, but the effectiveness of this approach is not yet clear and a better understanding of the relative importance of this pathway in various tumor settings is needed. We have evaluated syngeneic tumor models with different baseline inflammatory states to determine the contributions of STING activity in both tumor and non-tumor cellular compartments to anti-tumor immune responses. We find that productive anti-tumor immune responses in the poorly immunogenic B16F10 model show a strong dependence on STING expression in non-tumor cells. In the immunogenic MC38 model, constitutive STING activation in tumor cells can partially bypass the requirement for STING-dependent activity from immune cells. Our findings reveal multiple, context-dependent roles for STING activity in the regulation of anti-tumor immunity and the response to immunotherapy. In preclinical models where STING is basally active, checkpoint inhibition is more likely to have a therapeutic effect and removal of STING signaling from either the tumor or the non-tumor compartment has a minimal effect. Removal of STING signaling in both, however, diminishes the efficacy derived from checkpoint therapy. Further work is needed to understand the heterogeneity of STING signaling in patients, both in tumor cells and the tumor microenvironment, and the best means of harnessing this pathway to generate anti-tumor immunity and improve therapeutic outcomes.

## Introduction

Type I interferon (IFN) plays a critical role in promoting anti-tumor immune responses through pleiotropic stimulatory effects on both immune and non-immune cell populations [1]. Notably, type I IFN signaling in dendritic cell (DCs) has been shown to be critical for initiation of anti-tumor CD8+ T cell responses by promoting presentation of tumor-derived antigens and expression of pro-inflammatory cytokines and chemokines [2, 3]. While multiple upstream signals are capable of inducing type I IFN expression, evidence suggests the cyclic GMP-AMP synthase (cGAMP)/Stimulator of interferon genes (STING) DNA sensing pathway is the key mediator of this pathway [4, 5].

STING is activated following binding of cyclic dinucleotides produced by cGAS in response to cytosolic double-stranded DNA. STING activation results in the phosphorylation of TBK1 and production of type I IFN and downstream interferon stimulatory genes (ISGs) [6]. In tumor cells, genomic instability may lead to accumulation of cytosolic DNA that induces cGAS-dependent cGAMP production leading to tumor-intrinsic STING activation [7, 8]. In non-tumor cells, cGAMP released by tumor cells may activate STING in neighboring immune cells within the tumor microenvironment via the SLC19A1 transporter or gap junctions [9–11]. Alternatively, DNA may also be released from dying tumor cells, activating the cGAS/STING pathway in a tumor-extrinsic fashion [12]. Importantly, the relative contributions of tumor intrinsic and tumor-extrinsic STING activation leading to type I IFN production, and how that IFN production relates to tumor microenvironment inflammation and induction of productive anti-tumor immune responses, has not been well defined.

Here, we utilize two mouse tumor models with distinct immune phenotypes and immunotherapeutic intervention sensitivity to evaluate the varying roles of STING in regulating anti-tumor immunity. MC38 is a C57BL/6 origin colorectal cancer mouse syngeneic cell line that is characterized by robust immune infiltration and responsiveness to anti-PD1 therapy. In contrast, B16F10 is a C57BL/6 origin melanoma mouse syngeneic cell line with low immune cell infiltration and resistance to checkpoint inhibition [13]. Using mice and tumor cell lines deficient in STING signaling, we define cell context dependent roles for STING in regulating tumor inflammation and response to immune checkpoint blockade (ICB).

## Materials and methods

### Cell lines

The MC38-STING KO cell line was made through gene editing (Neon Transfection System, Invitrogen). Knockout was confirmed by western blot. Antibodies (Abs): TMEM173 (CST #13647S), β-actin (CST #3700S), anti-rabbit HRP (CST #7074). TMEM173 gRNA sequence: GTACCCAATGTAGTATGACC.

### Mice and tumor studies

5–8 week female C57BL/6J (000664) and C57BL/6J-TMEM173^gt^/J (017537) (Jackson Labs) were used. All experimental studies were conducted under protocols approved by the Institutional Animal Care and Use Committee of Amgen (IACUC). Animals were housed at Association for Assessment and Accreditation of Laboratory Animal Care (AAALAC) International-accredited facilities (at Amgen) in ventilated micro-isolator housing on corncob bedding. Animals had access ad libitum to sterile pelleted food and reverse osmosis-purified water and were maintained on a 12:12 h light:dark cycle with access to environmental enrichment opportunities. 2 × 10^5^ B16F10 (ATCC CRL-6475) or 3 × 10^5^ MC38 or MC38-STING KO cells were injected SC in the mice’ right flank. Tumor volume (mm^3^; LxWxH) was measured twice a week. Ab treatments, anti-CTLA4 (clone 9D9. 300ug/dose, mIgG2A) and anti-PD1 (clone 29F1A12 Cat# BE0273 in B16F10 studies and clone 29F1A12 mIgG1 N297G backbone in MC38 studies), and isotype antibodies (mIgG1 MOPC21, Cat#BE0083 and mIgG2a C1.18.4, Cat#BE0085 BioXCell) were given IP every 3 days for 3 doses starting at the time of randomization. Animals were randomized based on genotype and starting tumor volumes, typically 100mm^3^.

### Tumor isolation, flow cytometry

Tumors were digested in RPMI + 1% FBS containing 0.2 mg/ml Liberase TL, 20 U/ml DNase I (Roche Diagnostics). Cell staining: Live/Dead (Molecular Probes L23105), FcR-blocking (553142), and antibody cocktail (anti-B220 (RA3-6B2), anti-CD49b (DX5), anti-Thy1.2 (53-2.1), anti-CD25 (PC61), anti-TCRb (H57-597), anti-CD45 (30-F11), anti-CD4 (GK1.5), anti-CD8a (53-6.7), anti-FoxP3 (FJK-16s), Foxp3/transcription factor set (eBioscience 00-5523-00).

### Gene expression analyses

Tumors were flash-frozen in liquid nitrogen at indicated days post-implantation. *Fluidigm*: isolated RNA (Qiagen) were analyzed by Fluidigm (NC9872174) with a custom panel of immune lineage and interferon response gene probes. Gene expression was normalized to the geometric mean of 4 housekeeping genes using ΔCt. An interferon response gene signature was derived using the geometric mean of IFN-inducible genes (CMPK2, CXCL10, HERC5, IFIT1, IRF8, MX1, PD-L1). *Nanostring*: performed by nCounter XT Gene Expression Assay and the nCounter Mouse PanCancer Immune Profiling Panel gene codeset (SPRINT Profiler) and analysis (nSolver Analysis Software 4.0). *RNA sequencing*: cDNA library prepared in vitro cultured tumor cells. RNA sequencing reads (Illumina HiSeq platform) were aligned to mouse genome build 38 and transcript per million mapped reads were determined (Array Suite software (Omicsoft), in-house software). Ingenuity Pathway Analysis (IPA; Qiagen) was performed on genes with ≥ 4 expression in MC38 than B16 cells and top 5 canonical pathways by p value reported.

### In vitro assays

*ELISAs*: B16F10, MC38 or MC38-STING KO cells were plated at 1 × 10^6^ cells/ml in 100 μl. Transfected 2′3′-cGAMP (Invivogen), Poly(IC) (Invivogen), Interferon stimulatory DNA (ISD), or recombinant IFNβ (1 × 10^4^ U/ml) for 6 h with Lipofectamine (ThermoFisher). BX795 (TBK1) inhibitor (Invivogen) added at indicated concentrations. Cell supernatants were collected for mCXCL10 ELISA (abcam). ISD sequence and preparation: 5′-TACAGATCTACTAGTGATCTATGACTGATCTGTACATGATCTACA-3′ and its antisense oligonucleotides annealed at 95 °C and cooled to RT.

### Statistical analysis

Data were analyzed with GraphPad Prism7 software. P values were calculated by Mantel-Cox test for survival curves or one-way ANOVA with Bonferroni’s multiple comparisons test. Unpaired, two-tailed t-test was used for in vitro assays. *p < 0.05, **p < 0.01, ***p < 0.001, ****p < 0.0001.

## Results

### Requirement for STING expression in the non-tumor compartment for productive responses to ICB therapy varies across B16F10 and MC38 models

**Fig. 1:**
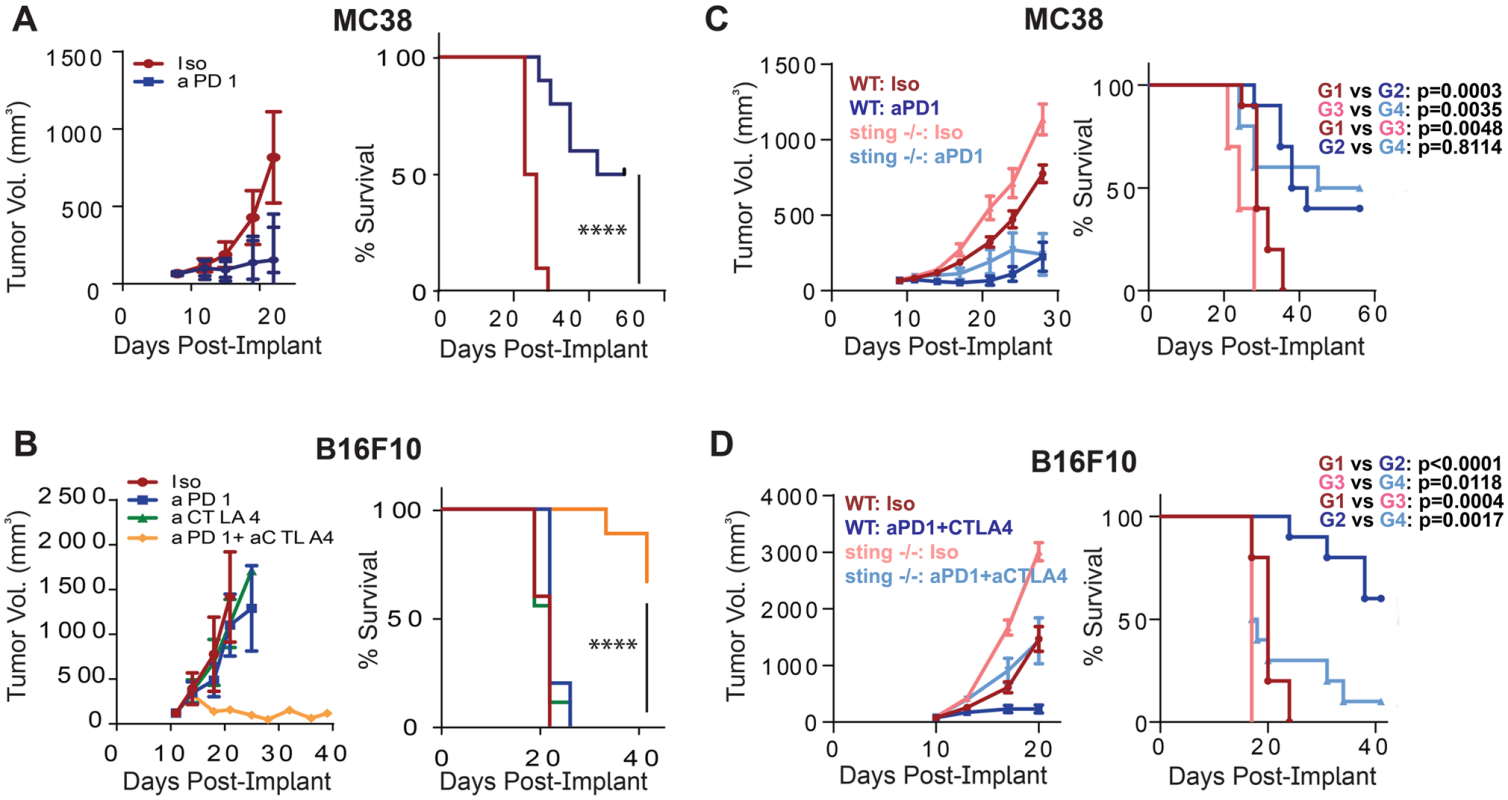
STING expression in non-tumor cells differentially regulates response to ICB in MC38 and B16F10 models. **A** Tumor growth in the MC38 tumor model with anti-PD1. **B** Effect of anti-PD1, anti-CTLA-4, or combination treatment on tumor growth in B16F10 model. **C** MC38 or **D** B16F10 tumors implanted in WT or STING KO (gt/gt) mice and treated as indicated. (*n* = 5–10 animals/group)

To investigate the role of STING expression by non-tumor cells during anti-tumor immune responses potentiated by ICB therapy, we established therapeutic treatment conditions in B16F10 melanoma and MC38 colon carcinoma tumor models that led to reproducible inhibition of tumor growth. We found that PD1 blockade alone was sufficient to reduce tumor growth in the MC38 model, while treatment with both anti-PD1 and anti-CTLA4 was required to inhibit growth of B16F10 tumors (Fig. 1A, B).

Utilizing these treatment conditions, we implanted wild-type or STING-deficient mice with B16F10 or MC38 tumors and evaluated the effect of ICB therapy on tumor growth. In both MC38 and B16F10 models, tumors grew faster in STING-deficient compared to wild-type animals (Fig. 1C, D), indicating a role for STING in regulating tumor growth under baseline conditions. However, surprisingly, in the MC38 model, ICB-mediated efficacy was similar between STING KO and wild-type animals (Fig. 1C). In contrast and consistent with previous findings [4], germline STING deficiency abrogated ICB-mediated efficacy in the B16F10 model (Fig. 1D). These data suggest that the role of the STING pathway in the non-tumor cell compartment may vary across different tumor types and that tumor-intrinsic factors may play a role in determining whether STING activity is required for the response to ICB therapy.

### Differential effect of STING expression between B16F10 and MC38 tumor models in the non-tumor compartment on the magnitude of the anti-tumor immune response

**Fig. 2:**
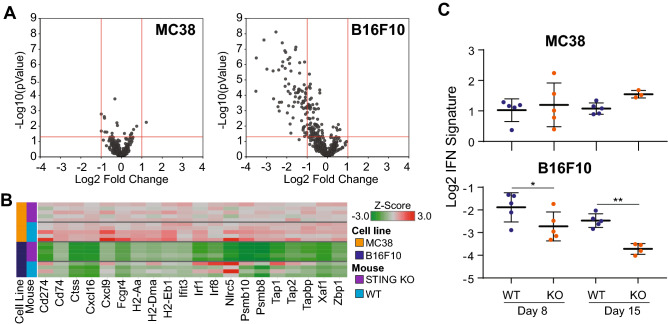
STING-dependent gene expression differences between MC38 and B16F10 tumors. **A** Volcano plots comparing gene expression changes in STING KO mice as a Log2 Fold change relative to wild-type mice in MC38 (left) and B16F10 (right) tumors, as determined by Nanostring. **B** Heatmap of top differentially expressed genes (|Log2 FC| ≥ 2; FDR ≤ 0.05) from (**A**). **C** Time course of IFN signature (CMPK2, HERC5, IFIT1, MX1, CD274, CXCL10) changes in B16F10 and MC38 tumors implanted into WT or STING KO mice and analyzed at day 8 or day 15 by Fluidigm

To further evaluate the dependence of anti-tumor immune responses on STING activity across different tumor models, we performed Nanostring analysis on whole MC38 or B16F10 tumors implanted in wild-type or STING-deficient mice and evaluated gene expression patterns. B16F10 tumors grown in STING-deficient animals had reduced expression of many genes compared to those grown in wild-type animals. In contrast, MC38 tumors showed minimal STING-dependent changes in gene expression (Fig. 2A). Consistent with the role of STING upstream of type I IFN production, the most differentially expressed genes from B16F10 tumors were enriched in known ISGs and immune-related genes (Fig. 2B). Comparison of the expression of these genes across tumor models revealed constitutively high ISG expression in MC38 tumors, with minimal effects of STING-deficiency, and lower expression in B16F10, but dramatic loss of expression in STING-deficient animals.

We extended our findings on differential STING-dependent IFN activity in B16F10 and MC38 models by performing a time course analysis of changes in IFN response genes in STING-deficient and wild-type animals using qRT-PCR. B16F10 tumors showed decreased expression of a composite IFN gene signature as early as day 8 post-implantation that became more apparent at day 15 post-implantation (Fig. 2C). In contrast, MC38 tumors showed a much higher baseline IFN gene signature that was unaffected by loss of STING in non-tumor cells. These data are consistent with MC38 being a more constitutively inflamed tumor model [14] and may relate to the increased sensitivity of MC38 tumors to anti-PD1 monotherapy relative to B16F10 tumors (Fig. 1A). Of note, B16F10 and MC38 tumors have different growth rates and as tumors increase in size, a lower fraction of the tumor derives from infiltrating immune cells such as dendritic cells that may strongly contribute to ISG expression. As the Nanostring data represents bulk tumor RNA analysis, the decreased proportion of these immune cells relative to other cells within the tumor microenvironment may lead to a larger difference in IFN signature score at later time points. Although in this study we did not specifically isolate the cellular source of the ISG expression, DC1s have been identified in the B16F10 model as the key producers of Type I IFN, mediated through STING [4]. The cell-intrinsic differences between B16F10 and MC38 tumors may lead to differential dependence on non-tumor STING activity to initiate and sustain anti-tumor immune responses.

### B16F10 and MC38 cells have intrinsic differences in type I IFN activity that affect ISG expression in vitro

**Fig. 3:**
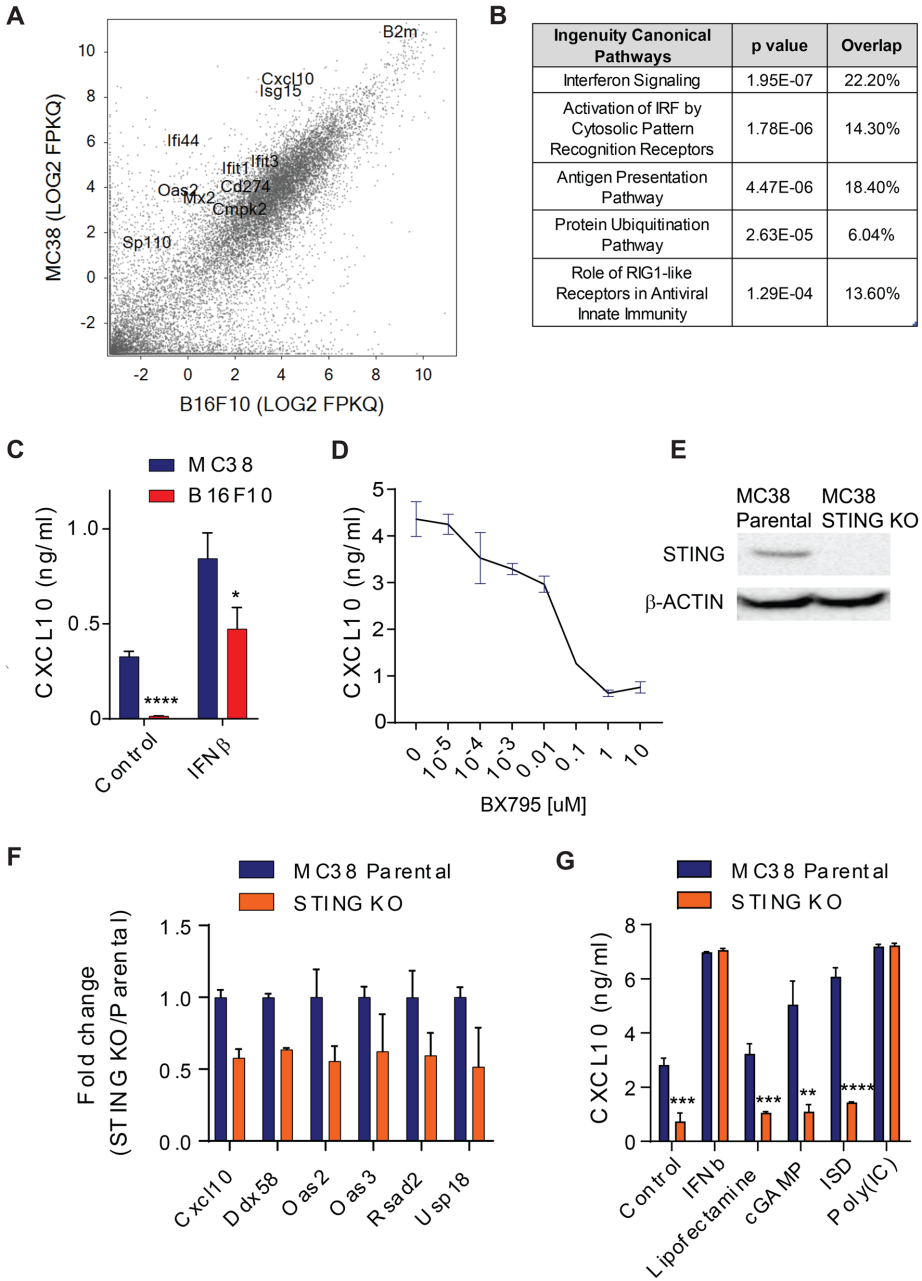
STING-dependent ISG activity in B16F10 and MC38 cells in vitro. **A** Comparison of gene expression in MC38 and B16F10 cultured tumor cells in vitro, as analyzed by RNAseq. **B** IPA analysis showing IFN and IFN-related pathways as top hits for genes preferentially expressed by MC38 compared to B16F10 cells. **C** CXCL10 secretion by MC38 and B16F10 tumor cell lines with or without IFNβ stimulation. **D** CXCL10 secretion by MC38 cells treated for 6 h with the TBK1 inhibitor, BX795, at indicated concentrations. **E** Western blot for STING and b-ACTIN in parental MC38 cells and MC38-STING CRISPR KO cells. **F** Type I IFN signature gene expression in MC38 parental and STING KO cell lines. **G** CXCL10 secretion by MC38 parental and STING KO cells treated with the indicated stimuli

We sought to define potential cell-intrinsic differences between B16F10 and MC38 tumors that underlie their differential dependence on STING activity in the non-tumor compartment. Comparing gene expression measured by RNA sequencing between in vitro cultured B16F10 and MC38 cells revealed the presence of several IFN responsive genes that were preferentially expressed by MC38 cells (Fig. 3A). Consistent with this finding, Ingenuity Pathway Analysis (IPA) on genes preferentially expressed by MC38 cells (≥ fourfold) demonstrated enrichment for pathways that are upstream and downstream of IFN signaling (Fig. 3B). Consistent with these RNA expression analyses, we also observed that CXCL10, a known IFN-responsive chemokine [15], was constitutively secreted by MC38 cells, but required type I IFN stimulation to be produced by B16F10 cells (Fig. 3C).

The above data raised the possibility that dysregulated activation of intracellular nucleic acid sensor signaling in MC38 cells leads to constitutive type I IFN pathway stimulation. To evaluate this hypothesis, we treated MC38 cells with an inhibitor of TBK1, BX795 [16], to block signaling downstream of STING and MAVS and observed a dose-dependent inhibition of CXCL10 production (Fig. 3D). Consistent with these findings, we also found that MC38 cells absent of STING expression (Fig. 3E) led to reduced expression of type I IFN response genes and CXCL10 protein production (Fig. 3F, G). Importantly, while STING KO MC38 cells lost response to cGAS/STING agonists, they were still responsive to stimulation with type I IFN and Poly(IC), a cGAS/STING-independent agonist of the RIG-I/MAVS and TLR3 pathways (Fig. 3G).

### Cell-intrinsic STING activity in MC38 cells promotes anti-tumor T cell responses and sensitivity to ICB

**Fig. 4:**
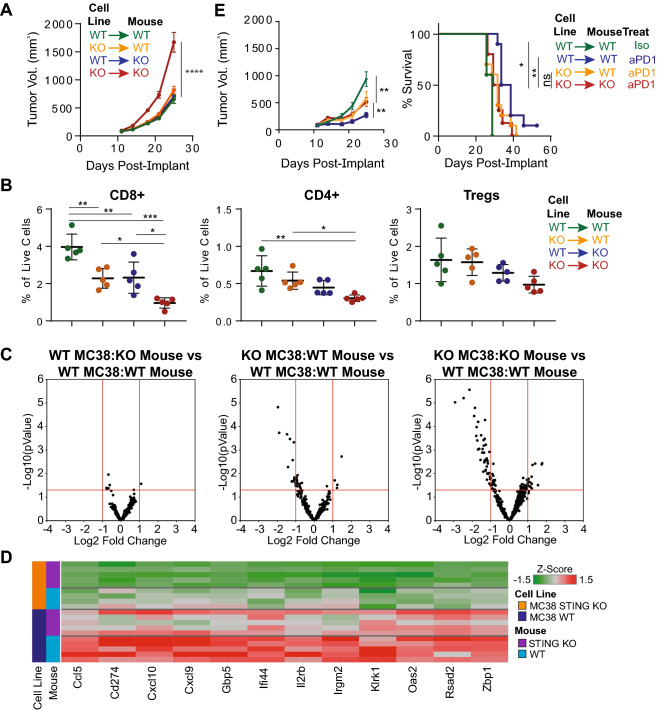
Absence of STING in MC38 tumor and non-tumor cells leads to dampened anti-tumor immunity. **A**–**D** Tumor volumes (**A**), Frequency of tumor-associated T cell subsets (**B**), gene expression changes as analyzed by Nanostring (**C**) and heatmap of top differentially expressed genes (|Log2 FC| ≥ 1; FDR ≤ 0.05) from wild-type or STING KO mice implanted with wild-type or STING KO MC38 tumor cells. For (**B**), all T cells are all gated on live cells, CD45^+^Thy1.2^+^TCRb^+^CD8^+^ for CD8^+^ T cells, or CD4^+^CD25^−^Foxp3^−^ for CD4^+^ cells and CD4^+^CD25^+^Foxp3^+^ for Tregs. **E** Tumor volumes and survival of wild-type or STING KO mice implanted with wild-type or STING KO-MC38 cells, with or without anti-PD1 treatment

We next evaluated the functional consequences of STING activation in MC38 tumor cells versus non-tumor cells on the host anti-tumor immune response by implanting wildtype or STING KO MC38 cells into wild-type or STING KO animals. We observed faster tumor growth when STING was absent in both tumor and non-tumor compartments (Fig. 4A), suggesting multiple sources of STING activity contribute to the anti-tumor immune response in this model. Consistent with these data, we observed reduced T cell infiltration into tumors when STING signaling was absent in either tumor or non-tumor cells, with the largest effect being seen when STING signaling was absent in both compartments (Fig. 4B). Interestingly, loss of STING activity appeared to have the greatest effect on CD8+ T cell infiltration into the tumor relative to CD4+ T cells or Tregs. Differential gene expression analysis from total tumor lysates demonstrated that a number of ISGs had decreased expression when STING activity was lost in non-tumor cells, but the dampened ISG expression was even more dramatic when STING was absent in both non-tumor and tumor compartments relative to wild-type conditions (Fig. 4C, D).

To determine whether the above differences in cell infiltration and gene expression had a functional consequence on the anti-tumor immune response, we treated STING wild-type or knockout animals implanted with STING wild-type or knockout tumor cells with ICB. In contrast to our earlier observations showing no effect of STING deficiency in the non-tumor compartment on ICB-mediated efficacy (Fig. 1C), when MC38 cells were deficient for STING, the magnitude of tumor growth inhibition following treatment was reduced (Fig. 4E). Taken together, these data suggest overlapping contributions of tumor and non-tumor STING activity in regulating the anti-tumor immune response.

## Discussion

We find that the cellular source of STING activation can dictate the importance of this pathway in regulating the anti-tumor immune response. The poorly immunogenic B16F10 mouse model has a strong dependence on STING expression in non-tumor cell types. In contrast, in the immunogenic MC38 model, constitutive STING activation in tumor cells can partially bypass the requirement for STING-dependent activity outside of the tumor cell compartment. Thus, a complex relationship likely exists between STING expression and downstream type I interferon responses in tumor cells and associated tumor stromal cell types, including immune cells, fibroblasts, and endothelial cells, that may dictate the baseline immune infiltration status of a tumor and its response to ICB therapy.

While the upstream signals responsible for constitutive STING activation in MC38 cells are unclear, pathways such as genomic instability [17], activation of endogenous viral elements [18], defects in endogenous nucleic acid degradation [19], or deregulation of STING itself [20] have been implicated in STING activation. Notably, neither TBK1 inhibition nor STING KO led to complete inhibition of constitutive type I IFN gene expression in MC38 cells, suggesting other pathways upstream or downstream of type I IFN signaling may also be dysregulated in these cells. Nevertheless, loss of STING activity in MC38 cells led to reduced tumor inflammation and partial loss of response to anti-PD1 treatment. Interestingly, only after STING signaling was abrogated in MC38 tumor cells did a contribution of STING signaling in non-tumor cell types become important to the anti-tumor immune response. These findings are consistent with dynamic crosstalk between tumor cells and the associated tumor microenvironment and potential negative feedback loops that could suppress STING signaling in one cellular compartment when signaling is high in another compartment.

Baseline type I IFN activity was substantially lower in the relatively non-immunogenic B16F10 model than in the MC38 model, both in in vitro cell culture and in vivo tumors. Lower overall levels of STING activation and downstream type I IFN signaling may be partially responsible for the resistance of B16F10 tumors to CPI monotherapy, and, based on our findings in the MC38 model, increasing STING activity in B16F10 tumor cells could reverse the resistance phenotype. Consistent with this hypothesis, STING agonists have shown improved efficacy in combination with PD1 blockade in multiple models, including B16F10 and KPC allografts [21–23]. Clinically, reduced expression of cGAS and STING in tumor cells correlates with poor survival in gastric cancer patients [24]. The benefit of these regimens, as indicated by data here, may depend on the compartments contributing to STING signaling and their crosstalk. Indeed, STING signaling in astrocytes was shown to promote tumor growth and metastasis [25].

The present study reveals diverse and context dependent roles of STING in regulating anti-tumor immunity and highlights the need to further define the contributions of nucleic acid sensing pathways in both tumor and non-tumor cell compartments to baseline tumor inflammation and response to immunotherapy. Whether direct STING agonism or molecules that increase DNA damage, such as PARP inhibitors [26, 27] or even chemotherapy [28], and lead to STING activation, is preferred requires further preclinical and clinical exploration and may be context dependent. In addition, single cell studies of the tumor microenvironment should clarify whether STING signaling is heterogeneous and subject to evolution – previous work has shown that epigenetic silencing of STING can occur [29] and this could be a biomarker for tumors that would respond differentially to STING agonists.

## References

[1] Tarbell KV, Egen JG (2018). Breaking self-tolerance during autoimmunity and cancer immunity: myeloid cells and type I IFN response regulation. J Leukoc Biol.

[2] Fuertes MB, Kacha AK, Kline J, Woo SR, Kranz DM, Murphy KM, Gajewski TF (2011). Host type I IFN signals are required for antitumor CD8+ T cell responses through CD8{alpha}+ dendritic cells. J Exp Med.

[3] Diamond MS, Kinder M, Matsushita H (2011). Type I interferon is selectively required by dendritic cells for immune rejection of tumors. J Exp Med.

[4] Woo SR, Fuertes MB, Corrales L (2014). STING-dependent cytosolic DNA sensing mediates innate immune recognition of immunogenic tumors. Immunity.

[5] Deng L, Liang H, Xu M (2014). STING-dependent cytosolic DNA sensing promotes radiation-induced type I interferon-dependent antitumor immunity in immunogenic tumors. Immunity.

[6] Dhanwani R, Takahashi M, Sharma S (2018). Cytosolic sensing of immuno-stimulatory DNA, the enemy within. Curr Opin Immunol.

[7] Ho SS, Zhang WY, Tan NY (2016). The DNA structure-specific endonuclease MUS81 mediates DNA sensor STING-dependent host rejection of prostate cancer cells. Immunity.

[8] Mackenzie KJ, Carroll P, Martin CA (2017). cGAS surveillance of micronuclei links genome instability to innate immunity. Nature.

[9] Marcus A, Mao AJ, Lensink-Vasan M, Wang L, Vance RE, Raulet DH (2018). Tumor-derived cGAMP triggers a STING-mediated interferon response in non-tumor cells to activate the NK cell response. Immunity.

[10] Luteijn RD, Zaver SA, Gowen BG (2019). SLC19A1 transports immunoreactive cyclic dinucleotides. Nature.

[11] Schadt L, Sparano C, Schweiger NA (2019). Cancer-cell-intrinsic cGAS expression mediates tumor immunogenicity. Cell Rep.

[12] Klarquist J, Hennies CM, Lehn MA, Reboulet RA, Feau S, Janssen EM (2014). STING-mediated DNA sensing promotes antitumor and autoimmune responses to dying cells. J Immunol.

[13] Homet Moreno B, Zaretsky JM, Garcia-Diaz A (2016). Response to programmed cell death-1 blockade in a murine melanoma syngeneic model requires costimulation, CD4, and CD8 T cells. Cancer Immunol Res.

[14] Kodumudi KN, Siegel J, Weber AM, Scott E, Sarnaik AA, Pilon-Thomas S (2016). Immune checkpoint blockade to improve tumor infiltrating lymphocytes for adoptive cell therapy. PLoS ONE.

[15] Dufour JH, Dziejman M, Liu MT, Leung JH, Lane TE, Luster AD (2002). IFN-gamma-inducible protein 10 (IP-10; CXCL10)-deficient mice reveal a role for IP-10 in effector T cell generation and trafficking. J Immunol.

[16] Clark K, Plater L, Peggie M, Cohen P (2009). Use of the pharmacological inhibitor BX795 to study the regulation and physiological roles of TBK1 and IkappaB kinase epsilon: a distinct upstream kinase mediates Ser-172 phosphorylation and activation. J Biol Chem.

[17] Bakhoum SF, Ngo B, Laughney AM (2018). Chromosomal instability drives metastasis through a cytosolic DNA response. Nature.

[18] Canadas I, Thummalapalli R, Kim JW (2018). Tumor innate immunity primed by specific interferon-stimulated endogenous retroviruses. Nat Med.

[19] Stetson DB, Ko JS, Heidmann T, Medzhitov R (2008). Trex1 prevents cell-intrinsic initiation of autoimmunity. Cell.

[20] Xia T, Konno H, Ahn J, Barber GN (2016). Deregulation of STING signaling in colorectal carcinoma constrains DNA damage responses and correlates with tumorigenesis. Cell Rep.

[21] Pan BS, Perera SA, Piesvaux JA (2020). An orally available non-nucleotide STING agonist with antitumor activity. Science.

[22] Nakamura T, Sato T, Endo R, Sasaki S, Takahashi N, Sato Y, Hyodo M, Hayakawa Y, Harashima H (2021). STING agonist loaded lipid nanoparticles overcome anti-PD-1 resistance in melanoma lung metastasis via NK cell activation. J Immunother Cancer.

[23] Ager CR, Boda A, Rajapakshe K (2021). High potency STING agonists engage unique myeloid pathways to reverse pancreatic cancer immune privilege. J Immunother Cancer.

[24] Song S, Peng P, Tang Z (2017). Decreased expression of STING predicts poor prognosis in patients with gastric cancer. Sci Rep.

[25] Chen Q, Boire A, Jin X (2016). Carcinoma-astrocyte gap junctions promote brain metastasis by cGAMP transfer. Nature.

[26] Ding L, Kim HJ, Wang Q (2018). PARP inhibition elicits STING-dependent antitumor immunity in Brca1-deficient ovarian cancer. Cell Rep.

[27] Pantelidou C, Sonzogni O, De Oliveria TM (2019). PARP inhibitor efficacy depends on CD8(+) T-cell recruitment via intratumoral STING pathway activation in BRCA-deficient models of triple-negative breast cancer. Cancer Discov.

[28] Fu G, Wu Y, Zhao G (2022). Activation of cGAS-STING signal to inhibit the proliferation of bladder cancer: the immune effect of cisplatin. Cells.

[29] Falahat R, Berglund A, Putney RM, Perez-Villarroel P, Aoyama S, Pilon-Thomas S, Barber GN, Mule JJ (2021). Epigenetic reprogramming of tumor cell-intrinsic STING function sculpts antigenicity and T cell recognition of melanoma. Proc Natl Acad Sci USA.

